# A comparative study of the performance of different large language models in the Chinese National Pharmacist Licensing Examination

**DOI:** 10.3389/fmed.2026.1880914

**Published:** 2026-07-06

**Authors:** Can Huang, Yanfang Sun, Wei Liu

**Affiliations:** Beijing Youan Hospital, Capital Medical University, Beijing, China

**Keywords:** artificial intelligence, large language models, pharmaceutical education, pharmacist licensing examination, standardized examination assessment

## Abstract

**Objective:**

To systematically evaluate the overall performance, subject-based differences, and question type adaptability of five mainstream large language models (ChatGPT, DeepSeek, Kimi, Qwen, and Doubao) in the Chinese National Pharmacist Licensing Examination (CNPLE), and to explore their feasibility as auxiliary tools for pharmaceutical examinations.

**Methods:**

A cross-sectional comparative study design was adopted. The practice questions of the 2024 CNPLE were used as the evaluation dataset, covering 480 standardized questions across four subjects: Pharmaceutical Professional Knowledge (I), Pharmaceutical Professional Knowledge (II), Comprehensive Knowledge and Skills of Pharmacy, and Pharmaceutical Administration and Regulation. The question types included Type A (single best choice), Type B (matching choice), Type C (comprehensive analysis), and Type X (multiple choice). Standardized prompts were used for independent tests using the official web versions of each model with default parameters. Each question was input separately in a new conversation session to avoid contextual interference. Taking the official standard answers as the gold standard, the subject accuracy rate, question type accuracy rate, and overall accuracy rate of each model were calculated. To compare the overall performance among the five models, Cochran’s Q test was applied. *Post-hoc* pairwise comparisons were performed using McNemar’s test with Bonferroni correction for multiple comparisons.

**Results:**

All five models exceeded the 60% passing score threshold of the CNPLE. The overall accuracy ranking was: Kimi (89.58%) > Doubao (88.96%) > DeepSeek (87.29%) > Qwen (77.92%) > ChatGPT (72.50%). Cochran’s Q test showed a statistically significant difference in the overall accuracy among the five models (*Q* = 111.39, df = 4, *P* < 0.001). Pairwise comparisons showed no significant differences among Kimi, Doubao and DeepSeek (*P* > 0.05), while all three performed significantly better than Qwen and ChatGPT (*P* < 0.001). At the subject level, all models achieved the best performance in Pharmaceutical Professional Knowledge (II) (average accuracy 89.50%) and relatively weak performance in Pharmaceutical Administration and Regulation (average accuracy 76.50%). At the question type level, Type C questions yielded the highest average accuracy (90.00%), whereas Type X questions had the lowest average accuracy (69.00%).

**Conclusion:**

In this single-run evaluation, the Chinese large language models tested achieved higher overall accuracy than ChatGPT under the same conditions. Among them, Kimi, Doubao and DeepSeek have reached an excellent performance level. Different models present differentiated advantages across subjects and question types. Regulatory subjects and Type X (multiple-answer) questions are common challenges for all models. The findings indicate that mainstream LLMs possess considerable potential as auxiliary tools for the CNPLE, and can provide intelligent support for pharmaceutical education and examination preparation.

## Introduction

In recent years, Large Language Models (LLMs), as a core application of generative artificial intelligence (AI), have made breakthrough progress in natural language understanding, knowledge reasoning, and content generation, with their applications in the healthcare sector expanding continuously (1–3). In the context of medical education, LLMs have proven capable of passing multiple high-difficulty standardized examinations, including the North American Pharmacist Licensure Examination (NAPLEX) (4), the Chinese National Medical Licensing Examination (CNMLE) (5), and the Chinese National Nursing Licensing Examination (CNNLE) (6), demonstrating great potential as intelligent teaching assistants, examination tutoring tools, and knowledge retrieval systems (7).

The Chinese National Pharmacist Licensing Examination (CNPLE) is a national licensing examination organized by the National Medical Products Administration (NMPA). It aims to evaluate whether pharmaceutical professionals possess the professional knowledge and skills required for practice, serving as a critical institutional safeguard to ensure public medication safety (8). The examination consists of four subjects: Pharmaceutical Professional Knowledge (I), Pharmaceutical Professional Knowledge (II), Comprehensive Knowledge and Skills of Pharmacy, and Pharmaceutical Administration and Regulation. It covers diverse fields such as medicinal chemistry, pharmaceutics, pharmacology, clinical pharmacotherapeutics, and drug administration regulations, assessing both basic theoretical knowledge and clinical practical application capabilities. Nevertheless, systematic research evaluating the performance of LLMs in the CNPLE remains insufficient. The performance differences, question type adaptability, and localization advantages of various models across different pharmaceutical subfields have not been fully clarified.

This study selects five mainstream Chinese and international large language models (ChatGPT, DeepSeek, Kimi, Qwen, and Doubao). Using the practice questions of the 2024 CNPLE as the evaluation benchmark, this paper systematically compares the performance of these models across different subjects and question types, and analyzes their strengths and limitations. The findings provide empirical evidence for the development of AI-assisted pharmaceutical education tools and the optimization of examination preparation modes for licensed pharmacists.

## Materials and methods

### Evaluation dataset

This study utilized a complete set of 480 official questions from the 2024 Chinese National Pharmacist Licensing Examination (CNPLE). To ensure the validity and standardization of the dataset, the questions were extracted from the 2026 edition of the National Licensed Pharmacist Qualification Examination Guidance Book (China Medical Science Press, March 2026, ISBN 978-7-5214-5865-7), which is the only officially designated and authorized preparation material for the CNPLE by the National Medical Products Administration (NMPA). The “2026” in the book title indicates its publication year, and its content consists of the full 2024 CNPLE questions and standard answers officially released by NMPA. The dataset covers all four core examination subjects, with 120 standardized multiple-choice questions allocated to each subject. All questions were manually typed into the testing system by two licensed pharmacist researchers, followed by full cross-verification to ensure 100% entry accuracy. No automated text extraction tools were used to avoid potential recognition errors.

We first conducted a random spot check of 30 sampled questions, and the results demonstrated that none of the evaluated large language models could reproduce the original question texts verbatim. To further assess the risk of public exposure, we subsequently performed exact matching and approximate semantic searches for all 480 questions across public question banks, online forums and social media platforms. We also implemented additional tests to examine whether the models could identify the source of these exam items. The results showed that no complete set of the 2024 CNPLE questions was disseminated on public networks, and all models failed to recognize the official source of the test questions. Detailed search methodology and quantitative results are provided in Supplementary Appendix S1. Nevertheless, we cannot fully rule out the possibility that the models were exposed to relevant pharmaceutical domain knowledge or fragmented exam content within their training corpora.

The detailed examination content of each subject is described as follows:

Pharmaceutical Professional Knowledge (I) (PPK I): This subject covers fundamental disciplines including medicinal chemistry, pharmaceutics, pharmacology, and pharmaceutical analysis. It primarily examines the chemical structures, physicochemical properties, preparation technologies, pharmacological mechanisms, and quality analytical methods of pharmaceuticals.

Pharmaceutical Professional Knowledge (II) (PPK II): Focusing on clinical pharmacotherapeutics, this subject involves 16 major categories of drugs, such as cardiovascular drugs, digestive drugs, endocrine drugs, and anti-infective drugs. The examined content includes clinical applications, indications, contraindications, adverse drug reactions, and drug interactions.

Comprehensive Pharmaceutical Knowledge and Skills (CPKS): This subject evaluates comprehensive pharmaceutical practice capabilities, including prescription review, drug dispensing, medication consultation, drug therapy management, prevention of medication errors, and the formulation of medication regimens for common diseases.

Pharmaceutical Administration and Regulation (PAR): This subject involves pharmaceutical policies and regulations, including the Pharmaceutical Administration Law, supervision of drug production and operation, professional ethics of licensed pharmacists, drug advertisement management, and controlled drug administration.

The CNPLE contains four types of test questions. The definitions and characteristics of each question type are specified below:

#### Type A: single choice questions

##### Definition

Each question consists of a stem followed by alternative options. There are four options for questions in the PAR subject and five options for the other three subjects, with only one optimal correct answer for each question.

##### Characteristics

This question type assesses basic knowledge and conceptual comprehension. Candidates need to examine questions carefully and capture key information to avoid loss of points caused by careless reading.

#### Type B: matching questions

##### Definition

A group of question stems shares one set of alternative options, which are placed before the stems. The options can be reused or unused, and each question has only one optimal answer.

##### Characteristics

This question type focuses on the relevance and matching of knowledge points. It requires rapid analysis of the correspondence between options and question stems, and is commonly used to examine drug efficacy and classification.

#### Type C: comprehensive analysis questions

##### Definition

Each question group contains contextual background information and 2–5 relevant sub-questions. These question groups are designed based on clinical scenarios and medical cases, and each sub-question has independent alternative options and one single optimal answer.

##### Characteristics

This question type evaluates comprehensive analytical capabilities. It requires analyzing each sub-question in the context of the background information, focusing on key details and logical relationships in the given cases.

#### Type X: multiple choice questions

##### Definition

Each question is composed of a stem and subsequent options. Four options are provided for PAR questions and five options for other subjects, with at least two correct answers. No points are awarded for over-selection, under-selection, or incorrect selection.

##### Characteristics

This question type has a relatively high difficulty level. It requires a solid command of professional knowledge and careful selection of options to prevent score loss from improper selection.

All test questions in this dataset consist solely of text, without any multimedia elements such as images or tables.

### Evaluation models

Five LLMs that are widely used in China were selected for evaluation in this study. All models were tested under default parameter settings without any domain-specific fine-tuning.

#### ChatGPT

Developed by OpenAI, the version used in this study is ChatGPT 5.4. It is one of the most widely used general-purpose large language models worldwide.

#### DeepSeek

Independently developed by DeepSeek Inc., the version used in this study is DeepSeek V3.2. It demonstrates strong capabilities in Chinese semantic understanding and complex logical reasoning.

#### Kimi

Launched by Moonshot AI, the version used in this study is Kimi 2.6. It offers powerful long-text parsing and accurate professional knowledge retrieval capabilities.

#### Qwen

Independently developed by Alibaba Group, the version used in this study is Qwen 3.5. It exhibits excellent multilingual adaptability and strong performance across multiple scenarios and tasks.

#### Doubao

Developed by ByteDance Inc., the version used in this study is Doubao 2.0. It performs well in daily Chinese interaction and professional question-answering across diverse fields.

#### Note

All models were accessed through public web interfaces with default parameter settings. Detailed testing environment information and model identifier clarifications are provided in Supplementary Appendix S2.

### Testing procedure

To ensure the objectivity and reproducibility of the evaluation results, a standardized testing procedure was implemented in this study:

#### Standardized prompting

A unified prompt in Chinese was applied to all models: “您正在参加中国执业药师资格考试。请仔细阅读以下题目, 并基于您的药学知识选择最佳答案。仅输出选项字母(A/B/C/D/E), 不要添加任何解释。” (English translation: You are currently participating in the Chinese National Pharmacist Licensing Examination. Please carefully read the following question and select the optimal answer based on your pharmaceutical knowledge. Only output the option letter (A/B/C/D/E) without any additional explanations.) This prompt required models to provide answers in the form of option letters and prohibited extra descriptions, but it did not limit the number of output letters. All models were allowed to generate two or more option letters for Type X multiple-answer questions.

#### Independent session testing

Each question was individually submitted in a brand-new blank conversation session to eliminate contextual interference with subsequent responses.

#### Test timing control

All model tests in this study were completed intensively from April 8 to April 9, 2026. To avoid interference from external variables including experimental environment and time span, and to ensure consistent test conditions as well as stable results for all models, researchers finished the evaluation of all test questions continuously and efficiently within these 2 days. This arrangement minimized the potential bias in model responses induced by temporal discrepancies. Therefore, the results reflect the performance of each model at this specific time point with a single response per question. No repeated testing or longitudinal tracking was performed.

#### Answer recording

The output answers of each model were manually recorded and compared with the official standard answers. Correct responses were marked as “1,” and incorrect responses were marked as “0.” For Type X multiple-answer questions, responses with only one single option letter, incomplete options, extra options or wrong options were all judged as incorrect in accordance with official exam scoring criteria (Supplementary Appendix S3).

#### Quality control

Question entry and answer verification were independently completed by two licensed pharmacist researchers within the study team to ensure data quality. To assess inter-rater agreement for manual answer recording, the two researchers independently transcribed the output letter (A/B/C/D/E) for all 2,400 model-question pairs. The raw agreement rate was 99.42% (2,386/2,400), and Cohen’s kappa was 0.994 (95%CI: 0.991–0.997), indicating almost perfect agreement. The 14 discordant cases were all due to minor typographical differences, all discrepancies were resolved through discussion.

The unified model settings and output processing rules are documented in Supplementary Appendix S2.

### Statistical analysis

Microsoft Excel 2021 was used for data collation, and SPSS 26.0 was used for data analysis. The overall accuracy rate, subject-specific accuracy rates, and question type-specific accuracy rates of each model were calculated. Because all five models answered the same set of 480 questions, the data have a paired binary structure (each response was scored as correct or incorrect). Cochran’s Q test was used for overall comparison of accuracy among the five models. *Post-hoc* pairwise comparisons were performed using McNemar’s test. Bonferroni correction was applied for multiple comparisons, with a correction factor of 10 (total pairwise comparisons for 5 models). For all pairwise comparisons, raw *P*-values from McNemar’s test are reported. Statistical significance was determined at Bonferroni-adjusted *P* < 0.005.

## Results

### Overall performance of each model

**FIGURE 1:**
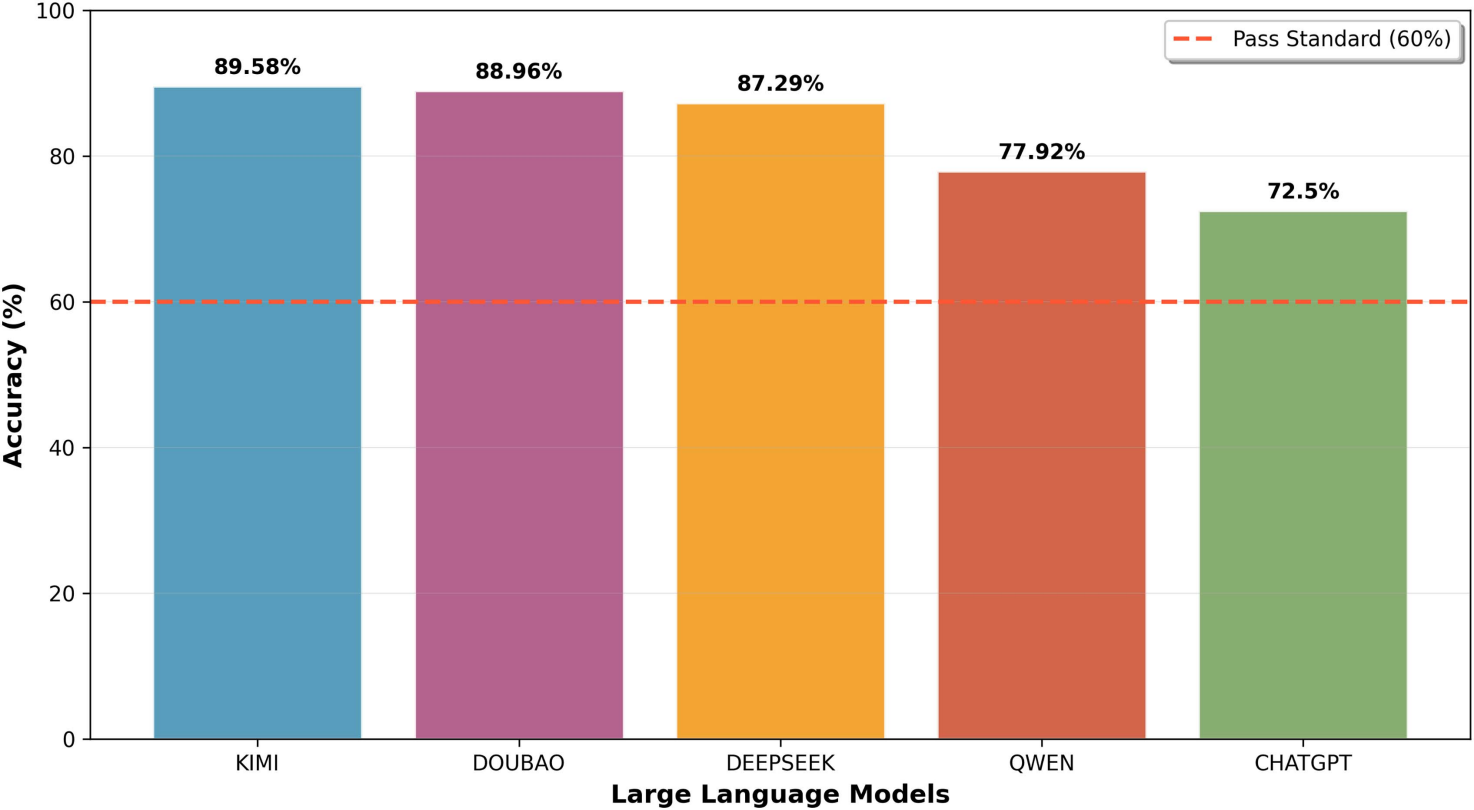
Overall accuracy comparison of each model in the CNPLE.

The overall performance of the five models is presented in Table 1. All models exceeded the passing threshold of 60% (Figure 1), indicating that each model possessed the fundamental capability to pass the CNPLE. Kimi achieved the highest overall accuracy rate of 89.58% in this single-run evaluation, followed by Doubao (88.96%) and DeepSeek (87.29%). These three models reached an excellent performance level ( ≥ 85%). By contrast, Qwen and ChatGPT exhibited relatively lower overall accuracy rates of 77.92 and 72.50%, respectively.

**TABLE 1:** Overall performance of each model in the CNPLE.

Model	Total correct answers	Total questions	Overall accuracy (%)	Rank	Pass status
Kimi	430	480	89.58	1	Passed
Doubao	427	480	88.96	2	Passed
DeepSeek	419	480	87.29	3	Passed
Qwen	374	480	77.92	4	Passed
ChatGPT	348	480	72.50	5	Passed

Cochran’s Q test showed a statistically significant difference in the overall accuracy among the five models (*Q* = 111.39, df = 4, *P* < 0.001) for this single-run testing session. *Post-hoc* pairwise comparisons using McNemar’s test with Bonferroni correction revealed no significant differences between Kimi and Doubao, Kimi and DeepSeek, or Doubao and DeepSeek (all *P* > 0.05). In contrast, each of these three models significantly outperformed Qwen and ChatGPT (all *P* < 0.001). Qwen showed a numerically higher accuracy than ChatGPT, but this difference did not reach statistical significance after Bonferroni correction.

### Differences in performance across subjects

The accuracy rates of each model across the four examination subjects are shown in Table 2. All models achieved the best performance in PPK II, with an average accuracy rate of 89.50%. Doubao and DeepSeek reached high levels of 95.00 and 93.33% in this subject, respectively. PAR was a common weakness for all models, with an average accuracy rate of 76.50%. ChatGPT obtained the lowest accuracy of only 65.83% in this subject.

**TABLE 2:** Accuracy rates of each model in different subjects-number correct/total (%).

Model	PPK I	PPK II	CPKS	PAR
ChatGPT	80/120 (66.67%)	98/120 (81.67%)	91/120 (75.83%)	79/120 (65.83%)
DeepSeek	108/120 (90.00%)	112/120 (93.33%)	104/120 (86.67%)	95/120 (79.17%)
Kimi	106/120 (88.33%)	112/120 (93.33%)	114/120 (95.00%)	98/120 (81.67%)
Qwen	106/120 (88.33%)	101/120 (84.17%)	76/120 (63.33%)	91/120 (75.83%)
Doubao	106/120 (88.33%)	114/120 (95.00%)	111/120 (92.50%)	96/120 (80.00%)
Mean	84.33%	89.50%	82.67%	76.50%

**FIGURE 2:**
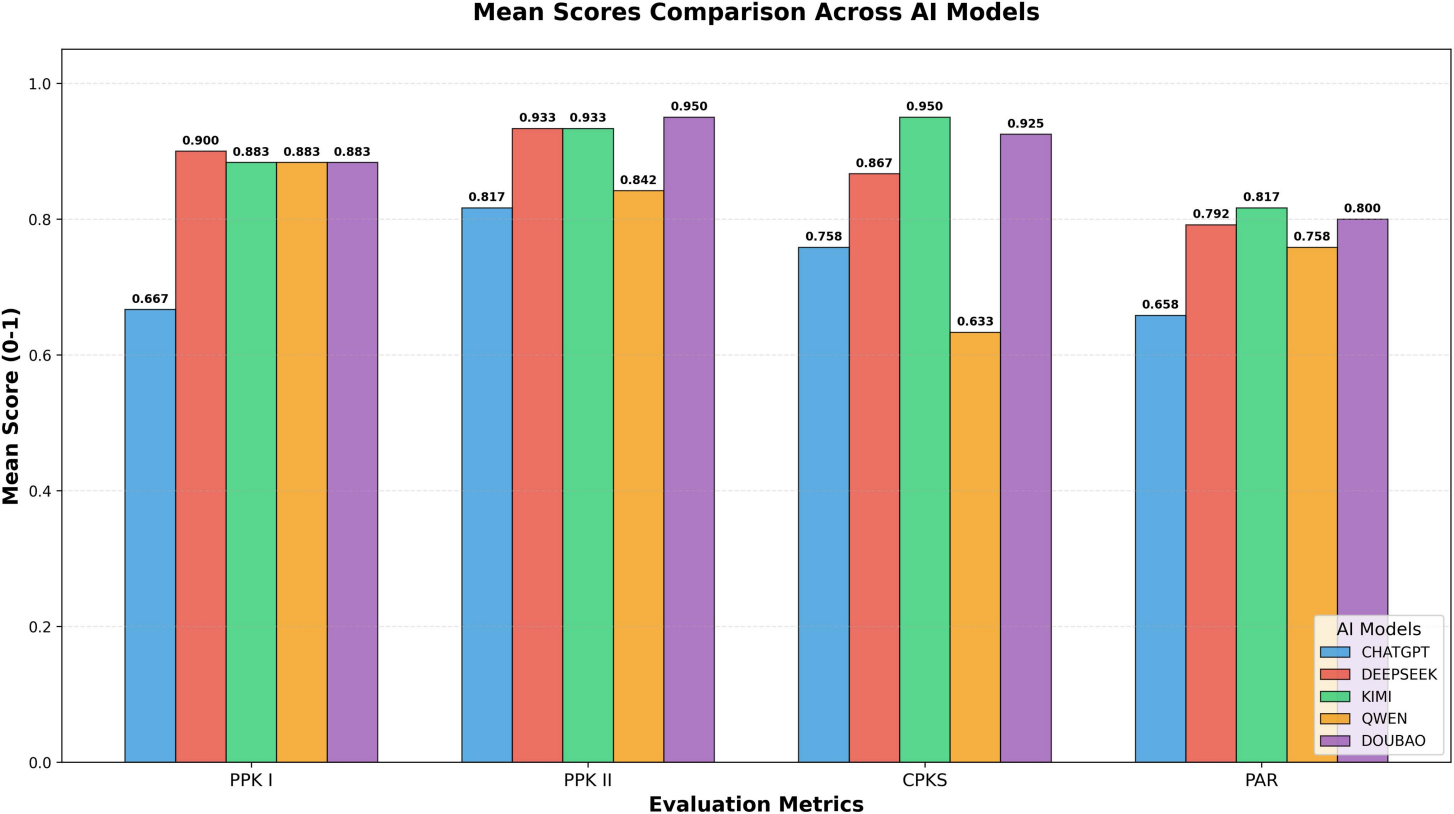
Performance of each model across the four examination subjects.

Model-specific performance is illustrated in Figure 2:

ChatGPT performed best in PPK II (81.67%) and worst in PAR (65.83%), showing substantial variation in performance across subjects.

DeepSeek achieved its highest accuracy in PPK II (93.33%) and the lowest in PAR (79.17%), with a consistently balanced performance across all subjects.

Kimi achieved an outstanding accuracy of 95.00% in CPKS, demonstrating strong comprehensive application capability in clinical pharmacy.

Qwen performed well in PPK I (88.33%) but showed a markedly lower accuracy in CPKS (63.33%), which represented a notable weakness.

Doubao achieved the highest accuracy rate among all models at 95.00% in PPK II, with balanced and excellent performance across all subjects.

### Differences in performance across question types

**FIGURE 3:**
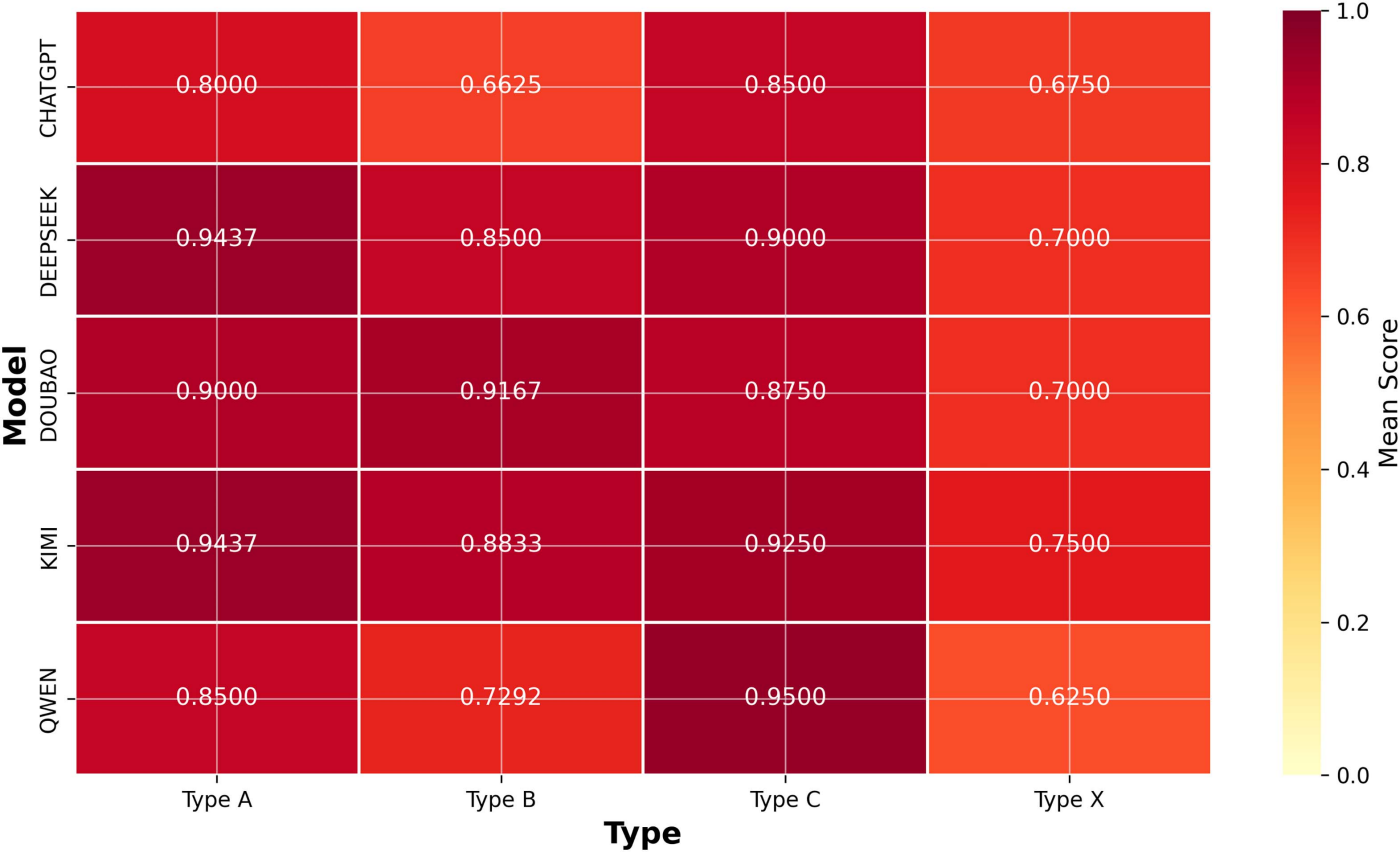
Heatmap of mean score by model and type.

The accuracy rates of each model for the four question types are presented in Table 3 and Figure 3. Type C questions (comprehensive analysis questions) yielded the best overall performance across models, with an average accuracy of 90.00%. Of note, Qwen achieved the highest accuracy of 95.00% for Type C questions, outperforming Kimi (92.50%) and DeepSeek (90.00%), indicating its particular strengths in case-based comprehensive reasoning.

**TABLE 3:** Accuracy rates of each model by question type-number correct/total (%).

Model	Type A (*n* = 160)	Type B (*n* = 240)	Type C (*n* = 40)	Type X (*n* = 40)
ChatGPT	128/160 (80.00%)	159/240 (66.25%)	34/40 (85.00%)	27/40 (67.50%)
DeepSeek	151/160 (94.38%)	204/240 (85.00%)	36/40 (90.00%)	28/40 (70.00%)
Kimi	151/160 (94.38%)	212/240 (88.33%)	37/40 (92.50%)	30/40 (75.00%)
Qwen	136/160 (85.00%)	175/240 (72.92%)	38/40 (95.00%)	25/40 (62.50%)
Doubao	144/160 (90.00%)	220/240 (91.67%)	35/40 (87.50%)	28/40 (70.00%)
Mean	88.75%	80.83%	90.00%	69.00%

Performance on Type A questions (single best choice) was second only to Type C. Both DeepSeek and Kimi reached a high accuracy of 94.38%. Doubao performed exceptionally well on Type B questions (matching questions), with an accuracy of 91.67%, reflecting strong capability in knowledge association and matching.

Type X questions (multiple-choice questions) were the most challenging for all models. No model achieved an accuracy above 75% for this question type, and the average across models was only 69.00%. Qwen performed the weakest on Type X questions in this single assessment, achieving merely 62.50%. However, this observation is based on only 40 Type X items (8.3% of the total dataset) and should therefore be interpreted as a preliminary finding requiring further validation with a larger sample of multiple answer questions.

## Discussion

### Overall advantages of Chinese large language models

In this single-run evaluation, Kimi, Doubao, and DeepSeek achieved significantly higher overall accuracy than ChatGPT under the same testing conditions. Qwen also showed numerically higher accuracy than ChatGPT, but this difference did not reach statistical significance after Bonferroni correction. Before presenting potential explanations for the observed accuracy differences, we emphasize that all such interpretations are speculative and hypothesis-generating rather than confirmatory. The training data, architectural details, and optimization strategies of commercial large language models are proprietary and not publicly disclosed. Consequently, we cannot establish causal links between model characteristics and performance outcomes. The following sections first report our empirical findings and then offer possible—but unverified—explanations that should be treated as working hypotheses requiring independent validation in future studies.

First possible hypothesis: differential exposure to Chinese-language pharmaceutical content. Although the exact composition of each model’s training corpus is not publicly disclosed, the observed performance differences might conceivably be associated with the proportion and quality of Chinese-language pharmaceutical content used during training. One speculative possibility is that Chinese models may have been exposed to a larger volume of Chinese pharmaceutical literature, textbooks, and examination-style materials, which might in theory facilitate a more accurate understanding of Chinese pharmaceutical terminology and better alignment with the CNPLE’s knowledge framework (9). In contrast, if ChatGPT was predominantly trained on English-dominated datasets, the relatively lower proportion of Chinese pharmaceutical content could hypothetically contribute to its weaker performance on China-specific regulatory and clinical questions (10). However, because the actual training data composition is undisclosed, this hypothesis cannot be tested with the current data.

Second possible hypothesis: localization optimization. Chinese models might have been optimized for Chinese language scenarios, which might offer a speculative explanation for their strong performance in processing long Chinese texts, answering professional knowledge questions, and conducting logical reasoning (11). For example, it is conceivable that Kimi’s reported long-text processing capabilities contributed to its performance on complex clinical cases in Type C questions, while Doubao’s strengths might similarly be hypothesized to relate to its excellent performance on knowledge-point matching in Type B questions. We stress that these are purely speculative associations, as our study design did not isolate or test specific architectural features.

Third possible hypothesis: domain-specific knowledge enrichment. Chinese developers have made substantial investments in healthcare AI (12), and one might hypothesize that collaborations with medical institutions and universities could have enriched the pharmaceutical knowledge base of these models. Such domain-specific enrichment might, if present, partially account for their improved performance in pharmaceutical examinations. Again, as the actual training data and optimization strategies are proprietary, these interpretations remain entirely speculative and require direct evidence from model developers to be verified.

### Analysis of disparities in subject performance

All models performed best in PPK II and relatively poorly in PAR, which can be explained by the following underlying factors.

PPK II mainly assesses the clinical application of various drugs, featuring clear knowledge points and rigorous logic. Relevant content is widely covered in pharmaceutical textbooks and literature. One speculative explanation for the strong performance across models in this subject is that such widely available content may be well represented in pre-training corpora, though this cannot be confirmed. Moreover, numerous questions in this subject are presented in the form of clinical cases, which might theoretically suit the pattern-recognition and reasoning capabilities of LLMs (13).

PAR is highly time-sensitive, with frequent content updates adjusted annually according to the latest laws, regulations, and policy documents. A plausible-but again unverified-explanation for the relatively lower performance in PAR is that the training data of LLMs inevitably have a time lag and may not fully incorporate the most recent regulatory updates (14). Several models failed to accurately master knowledge related to the newly revised Regulations for the Implementation of the Drug Administration Law included in the examination.

### Implications of disparities in question type performance

Accuracy differences across question types reflect the capability characteristics of LLMs.

Type A questions examine the memorization and comprehension of single knowledge points and represent the most adaptable question type for LLMs. All models achieved high accuracy rates, indicating their proficient mastery of basic pharmaceutical knowledge.

Type X questions require comprehensive command of knowledge points, with no points awarded for over-selection, under-selection, or incorrect selection, thereby placing stringent demands on the completeness and precision of knowledge. The unsatisfactory performance of LLMs on this question type is mainly attributable to model hallucinations, inaccurate demarcation of knowledge boundaries, and a tendency to mistakenly classify similar but incorrect options as correct. This remains a critical challenge restricting the application of LLMs in high-risk professional fields (15–17). It is also worth noting that some models were able to generate multiple option letters for Type X questions as requested by the prompt; however, because no prompt sensitivity analysis was conducted, we cannot definitively conclude that all models fully understood the multiple answer format, nor can we exclude the possibility that prompt design influenced performance on this question type. Moreover, given that only 40 Type X items were included, these observations should be considered preliminary.

Type C questions evaluate comprehensive analytical ability based on clinical cases. All models achieved outstanding performance on this question type, with an average accuracy rate of 90.00%, demonstrating the strong capacity of LLMs for clinical case comprehension and reasoning (18–20). This performance lays a foundation for their application in clinical pharmacy services.

### Advantages of large language models in the pharmaceutical field

The findings of this study demonstrate that LLMs exhibit multiple strengths in pharmaceutical practice.

First, LLMs possess powerful knowledge storage and retrieval capabilities, enabling rapid acquisition and integration of massive pharmaceutical knowledge (21). The outstanding performance of models in PPK II fully validates this advantage.

Second, LLMs are equipped with logical reasoning and problem-solving abilities. When answering comprehensive analysis questions, models can conduct rational reasoning and judgment by combining information provided in the question stem with acquired pharmaceutical knowledge to derive correct answers (22). This indicates that LLMs can not only memorize knowledge but also understand and apply it.

Additionally, LLMs have excellent natural language understanding and generation capabilities. They can accurately interpret complex medical terminology and pharmaceutical expressions, and generate clear, precise, and professional responses, laying a foundation for their application in pharmaceutical consultation and patient education (23, 24).

### Limitations of large language models in the pharmaceutical field

Despite the satisfactory performance of LLMs in this evaluation, their inherent limitations in pharmaceutical professional applications should be clearly recognized.

First, LLMs show relatively weak performance in questions involving complex chemical structures and drug action mechanisms, as reflected by their lower accuracy in PPK I. Content related to chemical structures and pharmacological mechanisms is highly abstract and specialized, requiring solid foundational knowledge in chemistry and biology, an area where the capability of current LLMs remains to be further improved.

Second, LLMs are prone to generating hallucinations-that is, plausible but factually incorrect responses (25). In this study, several models were found to fabricate non-existent drug names, dosages, or adverse reactions when answering obscure or detailed questions. This issue is particularly hazardous in pharmacy, as erroneous pharmaceutical information may pose severe threats to patients’ life and health (26).

Finally, LLMs lack genuine clinical experience. The work of licensed pharmacists requires not only solid theoretical foundations but also rich clinical experience to formulate individualized medication regimens according to patients’ specific conditions. However, LLMs can only reason based on existing knowledge and cannot accumulate clinical experience or handle complex clinical scenarios like human pharmacists.

### Application prospects of large language models in pharmacy

Despite the limitations discussed above, several research groups have begun exploring the potential use of LLMs in pharmaceutical education, clinical support, and regulatory tasks. However, it is important to emphasize that these applications remain largely experimental, and none of the following uses are yet ready for routine deployment without rigorous validation and human oversight.

In pharmaceutical education, preliminary studies suggest that LLMs may serve as auxiliary tools to provide personalized learning guidance (27–29). For example, they could generate targeted exercises or simulate examination-style questions. At present, such uses are best limited to controlled educational settings with instructor supervision.

In clinical pharmacy services, researchers have investigated whether LLMs could assist with prescription review, medication consultation, or patient education (30–34). While early results show promise, current LLMs are not reliable enough to be used independently in clinical practice. Any future implementation would require thorough validation against real-world cases and continuous oversight by licensed pharmacists.

In pharmaceutical supervision, some studies have explored the use of LLMs for tasks such as adverse drug reaction monitoring or package insert review (35–40). These are proof-of-concept investigations only; no regulatory authority has approved LLMs for such purposes. Substantial work remains to ensure accuracy, robustness, and compliance with legal standards.

In summary, while LLMs show potential in the pharmaceutical domain, their current capabilities are far from sufficient for autonomous real-world application. Any move toward practical deployment must be preceded by extensive validation, clear regulatory frameworks, and the active involvement of human experts.

## Limitations

First, the evaluation dataset was limited to the 2024 CNPLE questions, which cannot fully represent all pharmaceutical knowledge domains. Future studies should include questions from additional years and other pharmacy examinations.

Second, our results reflect only the specific model versions tested. As LLM technology evolves rapidly, future assessments are needed.

Third, a unified prompt was used for all question types, including Type X multiple-answer questions, which may have introduced response bias. Future research should optimize prompts for different question types.

Fourth, potential data contamination cannot be completely excluded. Although the 2024 CNPLE questions have not been publicly released, LLMs are trained on massive corpora containing pharmaceutical textbooks and similar materials. Overlap with test content is inevitable to a small degree.

Fifth, each question was tested only once. Web-based LLM outputs may vary due to server or model updates. Single-run testing cannot capture long-term response stability. Future studies using offline or locally deployed models should perform repeated testing.

Sixth, ChatGPT was accessed via a commercial VPN service due to regional access restrictions in China. This may affect access routes, regional policies, or available model versions, thereby limiting reproducibility of the ChatGPT-specific results. Future studies should use locally accessible, region-consistent interfaces to minimize such variability.

## Conclusion

This study systematically evaluated the performance of five mainstream LLMs in the CNPLE. The results indicated that all models could pass the examination. Among them, Chinese models including Kimi, Doubao, and DeepSeek achieved excellent performance in this single-run evaluation and overall superiority over ChatGPT, and their overall accuracy was higher than that of ChatGPT under the tested conditions. However, due to the single-time-point design, these findings should be considered preliminary and require replication with repeated testing. Different models present differentiated advantages across subjects and question types. Pharmaceutical Administration and Regulation and Type X (multiple-answer) questions represent common challenges for all evaluated models. The findings indicate the capability of LLMs in mastering and applying pharmaceutical professional knowledge, providing empirical evidence for their application in pharmaceutical education and examination tutoring. Future research could evaluate more models, optimize experimental conditions, expand evaluation dimensions, and explore the development and implementation of LLM-based intelligent pharmaceutical education systems.

## Data Availability

The original contributions presented in this study are included in this article/Supplementary material, further inquiries can be directed to the corresponding author.
