# Supplementary material for: A comparative study of the performance of different large language models in the Chinese National Pharmacist Licensing Examination
Source: Front Med (Lausanne). 2026 Jul 6;13:1880914. doi: 10.3389/fmed.2026.1880914 (PMC13383037; doi:10.3389/fmed.2026.1880914)
Supplement: Supplementary file 1 [file Table_1.docx]

Supplementary Appendix S1

Detailed Methodology and Results of Data Contamination Assessment

1. Search Platforms and Tools

| Category | Specific Platforms/URLs |
| --- | --- |
| General search engines | Bing ([https://www.bing.com](https://link.wtturl.cn/?target=https://www.bing.com&scene=im&aid=497858&lang=zh)), Baidu ([https://www.baidu.com](https://link.wtturl.cn/?target=https://www.baidu.com&scene=im&aid=497858&lang=zh)) |
| Medical examination question banks | National Medical Examination Network, Yikaobang, Medical Question Bank |
| Social media and forums | Zhihu ([https://www.zhihu.com](https://link.wtturl.cn/?target=https://www.zhihu.com&scene=im&aid=497858&lang=zh)), DXY Forum ([https://www.dxy.cn](https://link.wtturl.cn/?target=https://www.dxy.cn&scene=im&aid=497858&lang=zh)), Xiaohongshu ([https://www.xiaohongshu.com](https://link.wtturl.cn/?target=https://www.xiaohongshu.com&scene=im&aid=497858&lang=zh)) |
| Academic databases | China National Knowledge Infrastructure (CNKI), Wanfang Database |

2. Search Date

All searches were completed intensively on April 7, 2026, immediately prior to the formal model testing.

3. Search Strategy and Similarity Threshold

Exact phrase matching: The complete question stem (excluding option numbers) was entered directly into each search engine for exact phrase retrieval. A question was classified as "fully searchable" only if the identical complete stem appeared in the search results.

Approximate semantic matching: For each question, 3–5 core keywords were extracted and combined using Boolean logic for retrieval. A question was classified as "partially searchable" if the search result contained ≥60% continuous matching text from the original stem and retained complete core clinical information. For approximate semantic matching, we used a threshold of ≥60% continuous text overlap to identify any fragments that might contain related pharmaceutical knowledge; this broad cutoff was chosen as a conservative screening measure rather than as a definitive indicator of contamination.

4. Search Results Summary

| Search type | Fully searchable | Partially searchable | Unsearchable | Total |
| --- | --- | --- | --- | --- |
| Exact matching | 0 (0.00%) | 0 (0.00%) | 480 (100.00%) | 480 |
| Approximate semantic matching | 0 (0.00%) | 25 (5.21%) | 455 (94.79%) | 480 |
| Overall | 0 (0.00%) | 25 (5.21%) | 455 (94.79%) | 480 |

5.Platform‑by‑Platform Breakdown of Partially Matched Items (n=25)

| **Platform** | **Number of items** | **Typical form of matching** | **Options included?** | **Answers included?** | **Source nature** |
| --- | --- | --- | --- | --- | --- |
| Zhihu | 11 | Fragmented keyword mentions in user questions/discussions | No | No | Secondary dissemination (user‑generated content) |
| DXY Forum | 9 | Isolated drug/disease names in study notes or clinical discussions | No | No | Secondary dissemination (user‑generated content) |
| Xiaohongshu | 5 | Brief topical references in exam preparation posts | No | No | Secondary dissemination (user‑generated content) |
| **Total** | **25** | – | **0** | **0** | – |

6. Additional Explanation

All 25 partially searchable questions only involved basic knowledge points of common diseases . None of them contained the complete original question stem, interference option design or standard answer combinations used in this study.

7.Sensitivity analysis comparing partially searchable vs. unsearchable questions

we performed a post‑hoc sensitivity analysis. For each of the five models, we calculated the accuracy on the 25 partially searchable questions and on the remaining 455 unsearchable questions. The accuracy differences between the two groups were minimal (≤3.5 percentage points) and not statistically significant for any model. Notably, in some models the partially searchable questions actually had slightly lower accuracy, which does not support the notion that partial semantic overlap conferred any measurable advantage. These findings strongly suggest that the 25 flagged items did not contribute to inflated model performance.
