# Supplementary material for: A comparative study of the performance of different large language models in the Chinese National Pharmacist Licensing Examination
Source: Front Med (Lausanne). 2026 Jul 6;13:1880914. doi: 10.3389/fmed.2026.1880914 (PMC13383037; doi:10.3389/fmed.2026.1880914)
Supplement: Supplementary file 2 [file Table_2.docx]

**Supplementary Appendix S2**

| Platform Display Name (Main Manuscript) | Version | Developer | Version Type | Interface Type |
| --- | --- | --- | --- | --- |
| ChatGPT 5.4 | gpt-5.4-mini | OpenAI | Free public version | Official web interface |
| DeepSeek V3.2 | deepseek-chat | DeepSeek Inc. | Free public version | Official web interface |
| Kimi 2.6 | kimi-k2 | Moonshot AI | Free public version | Official web interface |
| Qwen 3.5 | qwen3.5-plus | Alibaba Group | Free public version | Official web interface |
| Doubao 2.0 | doubao-seed-2-0-pro | ByteDance Inc. | Free public version | Official web interface |

**1. Basic Information of Evaluated Large Language Models**

**2. Access Information & Testing Environment**

1. **Unified Testing Period**: April 8, 2026 – April 9, 2026
2. **Testing Region**: Mainland China
3. **Official Access URLs & Access Methods**

- ChatGPT 5.4 mini: <https://chat.openai.com>
- DeepSeek V3.2: <https://www.deepseek.com>
- Kimi 2.6: <https://kimi.moonshot.cn>
- Qwen 3.5: <https://tongyi.aliyun.com>
- Doubao 2.0: <https://www.doubao.com>

**Access Mode for All Models**: Pure web interface access; no API, third-party clients or modified tools were used.

**3. Unified Functional Settings (All Models)**

To maintain consistent experimental conditions, all extended auxiliary functions were **permanently disabled** during the entire test:

- Web search function: Disabled
- Deep reasoning / Chain-of-Thought mode: Disabled
- Third-party plugins: Disabled
- File upload & parsing function: Disabled

**4. Parameter Settings**

All decoding parameters (including temperature, top_p, maximum output length, and frequency penalty) were set to the platform default values. These parameters were not visible or controllable to users through the public web interfaces. No custom parameter adjustments were made during the experiment.

**5. Unified Rules for Non-standard Output Processing**

All model responses were scored following the identical criteria below:

1. Standard output (only single/multiple option letters A/B/C/D/E as required): Recorded as valid answers directly.
2. Output containing extra explanatory text but clear option letters: Only the option letters were extracted for scoring; redundant content was ignored.
3. Output without any valid option letters: Marked as incorrect (0 point).
4. Type X multiple-choice questions (at least two correct answers): Answers containing all standard correct options were judged correct; answers with missing, partial or wrong options were judged incorrect (no partial credit).

**6. Conversation Record Management**

All original chat logs between researchers and models were stored in local encrypted files for internal data verification.
